# Anti-CD8 monoclonal antibody-mediated depletion alters the phenotype and behavior of surviving CD8+ T cells

**DOI:** 10.1371/journal.pone.0211446

**Published:** 2019-02-08

**Authors:** Eric W. Cross, Trevor J. Blain, Divij Mathew, Ross M. Kedl

**Affiliations:** Department of Immunology and Microbiology, University of Colorado, Denver, Colorado, United States of America; Saint Louis University School of Medicine, UNITED STATES

## Abstract

It is common practice for researchers to use antibodies to remove a specific cell type to infer its function. However, it is difficult to completely eliminate a cell type and there is often limited or no information as to how the cells which survive depletion are affected. This is particularly important for CD8+ T cells for two reasons. First, they are more resistant to mAb-mediated depletion than other lymphocytes. Second, targeting either the CD8α or CD8β chain could induce differential effects. We show here that two commonly used mAbs, against either the CD8α or CD8β subunit, can differentially affect cellular metabolism. Further, *in vivo* treatment leaves behind a population of CD8+ T cells with different phenotypic and functional attributes relative to each other or control CD8+ T cells. The impact of anti-CD8 antibodies on CD8+ T cell phenotype and function indicates the need to carefully consider the use of these, and possibly other “depleting” antibodies, as they could significantly complicate the interpretation of results or change the outcome of an experiment. These observations could impact how immunotherapy and modulation of CD8+ T cell activation is pursued.

## Introduction

Few scientific discoveries have had as much of an impact on the biological sciences as the generation of antibodies against specific molecules of interest, particularly the advent of the means to generate monoclonal antibodies (mAb) using hybridomas. The specificity and affinity innate to mAbs created a means to: robustly delineate and classify types of cells and their lineage, reliably assay for molecules of interest *in vitro* and *ex vivo*, remove cell types from an organism, increase specificity of drug delivery, and influence signaling by cross-linking target molecules or acting as an agonist/antagonist directly. Unfortunately, a particular mAb does not accomplish these feats in isolation. As an example, a mAb utilized to deliver an attached therapeutic to a particular cell type may complicate the intended goal by physically impeding binding of the target to its normal binding partners or promote activation of the target by stabilizing the target’s interaction with its ligand. Either scenario could impact the delivered drug’s efficaciousness. Hopefully, these scenarios would be studied and likely become evident if the mAb is to be used clinically, however, as a tool for basic/pre-clinical research we are often ignorant of the unintended consequences of these reagents.

Monoclonal antibodies have been used for decades with certain assumptions of their behavior, yet unintended consequences of their use discovered much later. As an example, both anti-Asialo-GM1 and anti-NK1.1 mAbs deplete natural killer cells from the blood efficiently, however, only anti-NK1.1 removes these cells from peripheral tissues effectively [1]. As blood is frequently used as a convenient proxy to determine depletion efficacy, it can appear that all natural killer cells are depleted after anti-Asialo-GM1 treatment when they are not. Anti-Asialo-GM1 has also been found to remove basophils, another unintended consequence of its use [2]. Similarly, the anti-Gr1 mAb, commonly used to deplete neutrophils, has only recently been shown to remove memory CD8+ T cells that could complicate the interpretation of results [3]. This is because CD8+ T cells, or cytotoxic T lymphocytes, act as an effector cell, like neutrophils, but control pathogens by differing means. CD8+ T cells directly lyse or induce apoptosis of cells presenting cognate, foreign peptides. The mechanism of T cell activation becomes particularly important when considering other effects or targets of antibody treatment. Besides depleting cells, certain mAbs have demonstrated immunomodulatory activity by altering how a T cell perceives an activating signal. Peptide loaded onto MHC-I (peptide-MHC-I) are recognized as a complex by the T cell receptor of CD8+ T cells and the associated complex of CD3 polypeptides that initiate the signaling cascade, a CD8+ T cell undergoes activation and differentiation when an innate antigen-presenting cell presents the cognate antigen in the proper inflammatory context. In mice, the CD3 signaling complex was recently successfully manipulated by a mono Fab anti-CD3ε therapy is able to boost the activation of antigen-specific CD8+ T cells and results in decreased tumor burden [4]. Further, using human CD8+ T cell lines it has been shown that certain anti-CD8 mAb clones can increase the threshold of activation whereby only pathogen-specific T cells could respond, but autoimmune T cells could not [5]. This was presumed to be due to mAb-mediated blockade of CD8 binding to MHC-I and destabilizing the TCR:peptide-MHC-I interaction. Due to the potential dual use of mAbs as signaling modulators as well as means to deplete a cell type, in addition to increased use of mAbs clinically, we asked whether these reagents could affect the behavior of cells that fail to be removed. This could prove an important aspect to consider given that much data has been generated using such reagents with the assumption that the cell type in question is no longer in play.

The use of depleting mAb reagents most successfully in the clinic will require an understanding of how the remaining population is altered. This is already a concern in the case of transplant rejection, which in part is caused by homeostatic expansion of surviving T cells after depletion [6]. Here we chose to focus on CD8+ T cells for two reasons. First, there are two main target molecules for depletion, CD8α and CD8β that could result in differences in the behavior of the survivors. Second, the mAbs commonly used to deplete CD8+ T cells often leave behind a population amenable to study and hence could alter how results are interpreted [7–11]. Herein we demonstrate that treatment with the commercially available anti-CD8α mAb, clone 53–6.7, and anti-CD8β, clone 53–5.8, mAbs result in a population of surviving CD8+ T cells that can participate in an immune response. At the peak of the response, CD8+ T cells surviving either anti-CD8α or –β mAb display differences in differentiation markers compared to each other and control, untreated CD8+ T cells. Additionally, homing markers differ and are concurrent to a striking difference in localization within the spleen between anti-CD8α or –β mAbs. Further, these mAbs affect cytotoxic function similarly when added to *in vitro* stimulated CD8+ T cells at the time of the assay, yet differentially alter the cytotoxic function of depletion-surviving CD8+ T cells after treatment and activation *in vivo*. Lastly, we demonstrate that anti-CD8α mAb is uniquely able to boost both glycolysis and mitochondrial respiration relative to untreated CD8+ T cells when stimulated *in vitro*, potentially suggesting a mechanism behind the differences shown. These results have a potential impact on experiments whose interpretation assumes the absence of CD8+ T cells and also hint at how direct CD8 stimulation effects CD8+ T cells with potential applications for immunotherapy.

## Methods

### Ethics statement

Housing and care of laboratory animals was conducted according to the National Institutes of Health guidelines for the housing and care of laboratory animals. All animals were observed daily by Office of Laboratory Animal Resources (OLAR) of the University of Colorado Denver animal care staff for health related concerns and the veterinary staff was contacted if a concern was identified. All experiments involving mice were conducted following protocols approved by the Institute of Animal Care and Use Committees (IACUC) of the University of Colorado Denver. Mice were humanely euthanized by carbon dioxide inhalation followed by cervical dislocation.

### Mice and reagents

All experiments were performed with 5–10 week old C57BL/6 mice of either sex, purchased from Jackson Laboratories. OT1 are a TCR transgenic mouse specific for the SIINFEKL peptide derived from ovalbumin (amino acids 257–254) in the context of H-2K^b^ and were bred and housed at the University of Colorado vivarium. All animal protocols were approved by the Institute of Animal Care and Use Committees of the University of Colorado. Antibodies used for depletion, anti-CD8α (clone 53–6.72) and anti-CD8β (clone 53–5.8), were purchased from Bio X Cell and/or made in house. Fluorochrome-conjugated antibodies used for flow cytometry include CD8α, CD8β, B220, CD44, CD62L, MHC-II, CD45.1, CD45.2, CD122, CD127, Eomesodermin and KLRG1 all purchased from Biolegend and Goat anti-Rat IgG from Jackson Immunoresearch Laboratories. Fluorochrome-conjugated antibodies used for microscopy include MOMA-1 (BioRad), IgM (in house), and CD3ε and CD45.1 (Biolegend). SIINFEKL and SIYNFEKL peptide used for *in vitro* stimulation or *in vivo* vaccination was synthesized by the University of Colorado Protein Production Shared Resource facility.

### OT1 adoptive transfer assays and assessing depletion-surviving CD8+ T cell phenotype and function

OT1 T cells were isolated from whole splenocytes by CD8-negative magnetic selection (Biolegend) and 10^6^ cells were adoptively transferred, unless otherwise noted, into CD45-congenic recipient mice by tail vein injection. The following day 250–500μg of depleting antibody was delivered intraperitoneally. For subunit-vaccinations, 100μg whole ovalbumin (Sigma), 50μg poly(I:C) (Sigma), and 50μg anti-CD40 (clone FGK4.5, made in house or from BioXCell) suspended in PBS was given intravenously and assessed 7 days later unless otherwise stated. For infectious challenge, 10^7^ PFU of Vaccinia virus expressing ovalbumin was given intravenously and assessed 5 days later unless otherwise stated. Spleens and lymph nodes harvested were macerated with glass slides, RBC lysed with ACK buffer, and stained with fluorochrome-conjugated antibodies to determine phenotype of transferred OT1 T cells.

### Confocal microscopy

For imaging, spleens and lymph nodes were harvested from mice and fixed on ice for 30min in 1% PFA with 3% sucrose in PBS. Tissue was subsequently incubated on ice with 20% sucrose in PBS for 30-60min. Tissue samples were then frozen in OCT media using dry ice. A Leica Cryostat was used to cut 5–7μm sections for staining. Sections were imaged using a Zeiss LSM 700 confocal microscope at x10 magnification. Images were analyzed using Imaris or Zen Blue software. For quantification, the white pulp was delineated by IgM and MOMA-1 staining, while the red pulp was identified by the absence of staining. CD45.1+ cells within that staining were then considered white pulp resident, whereas those outside were red pulp resident.

### Cytotoxicity assays

OT1 T cells were isolated, adoptively transferred, and the host mice treated with depleting mAbs as before. Spleens and lymph nodes were harvested and CD8+T cells purified by CD8-negative selection as described above. OT1 T cells were then isolated by flow-assisted cell sorting by CD45-congenic staining. OT1 T cells were then added to cultures of B16 tumor cells expressing RFP in a 96-well plate at a 1:1 effector to target ratio. Incucyte active caspase-3/7 dye was added to the media as a means to quantify dying cells. Plates were then imaged using the IncuCyte ZOOM (EssenBioscience). Curves of the total death or ratio of dead to live tumor target cells over time were generated by quantifying 2–4 fields/well taken every 1-2hours. To assess the impact of mAbs on TCR:peptide-MHC-I binding we cultured OT1 splenocytes with SIINFEKL peptide (2μg/mL) for 2 days followed by washing and resuspended the splenocytes in media supplemented with IL-2 (25U/mL) for 3 days. For the IncuCyte assay, OT1 T cells were isolated using the Biolegend CD8 negative magnetic selection kit at plated at an effector to target ratio of 10:1. Anti-CD8 mAbs were then added to the media at various concentrations for the cytotoxicity assay.

### Metabolic flux assay

CD8+ T cells from unmanipulated mice were purified using a BioLegend CD8 negative magnetic selection kit and plated at 10^6^/well on a 24-well plate previously coated overnight with anti-CD3ε (clone 145-2C11; Tonbo Biosciences) at 1μg/mL in PBS. Costimulation was provided in the media at 1μg/mL with anti-CD28 (clone 37.51; BioLegend). Either anti-CD8α or –β were added to the media at 10ng/mL. The CD8+ T cells were harvested 2–3 days later, seeded at equal numbers per well onto a 96-well poly-D-lysine coated Seahorse plate in Seahorse XF RPMI with L-glutamine with or without dextrose and equilibrated for 1hr at 37C and 0%CO_2_. Oxygen consumption rate and extracellular acidification rate were measured using a Seahorse XFe96 analyzer (Agilent Technologies) following the manufacturer’s instructions in the Seahorse XF Mito Stress Test manual. Briefly, oligomycin (ATP-synthase inhibitor; final concentration (FC) = 1μM), FCCP (uncouples the mitochondrial membranes; FC = 1μM), and antimycin A + rotenone (electron transport chain inhibitors; FC = 0.5μM each) were injected sequentially into wells with dextrose containing media to determine measures of respiration. Similarly, cells seeded into glucose free media were assayed for glycolytic measures throughout injections of dextrose (10mM), oligomycin (ATP-synthase inhibitor; FC = 1μM), and 2-deoxyglucose (glycolysis inhibitor; FC = 50mM). All drugs were provided as part of manufacturer kits or purchased from Sigma and diluted in Seahorse XF media as recommended in the Seahorse XF Mito Stress Test manual.

### Statistical analyses

Graphpad Prism 7 software was used for all statistical analyses. The Student’s two-tailed, unpaired t-test was used for splenic localization data, comparing depleting-antibody treatment groups to each other, and for metabolic flux data, comparing depletion-antibody treatment groups to control. For all other data one-way ANOVA was applied and if a significant difference was determined multiple-comparisons of means was used to generate a p-value. For cytotoxicity experiments, one-way ANOVA was applied to the area under the curve values generated from the time course curves. All error bars represent mean +/-SEM. * = p<0.05, ** = p<0.01, *** = p<0.001, **** = p<0.0001, non-significant differences were not displayed for simplicity.

## Results

### CD8+ T cells that survive depletion by mAb treatment differentially internalize CD8 and retain mAb on their surface

**Fig 1:**
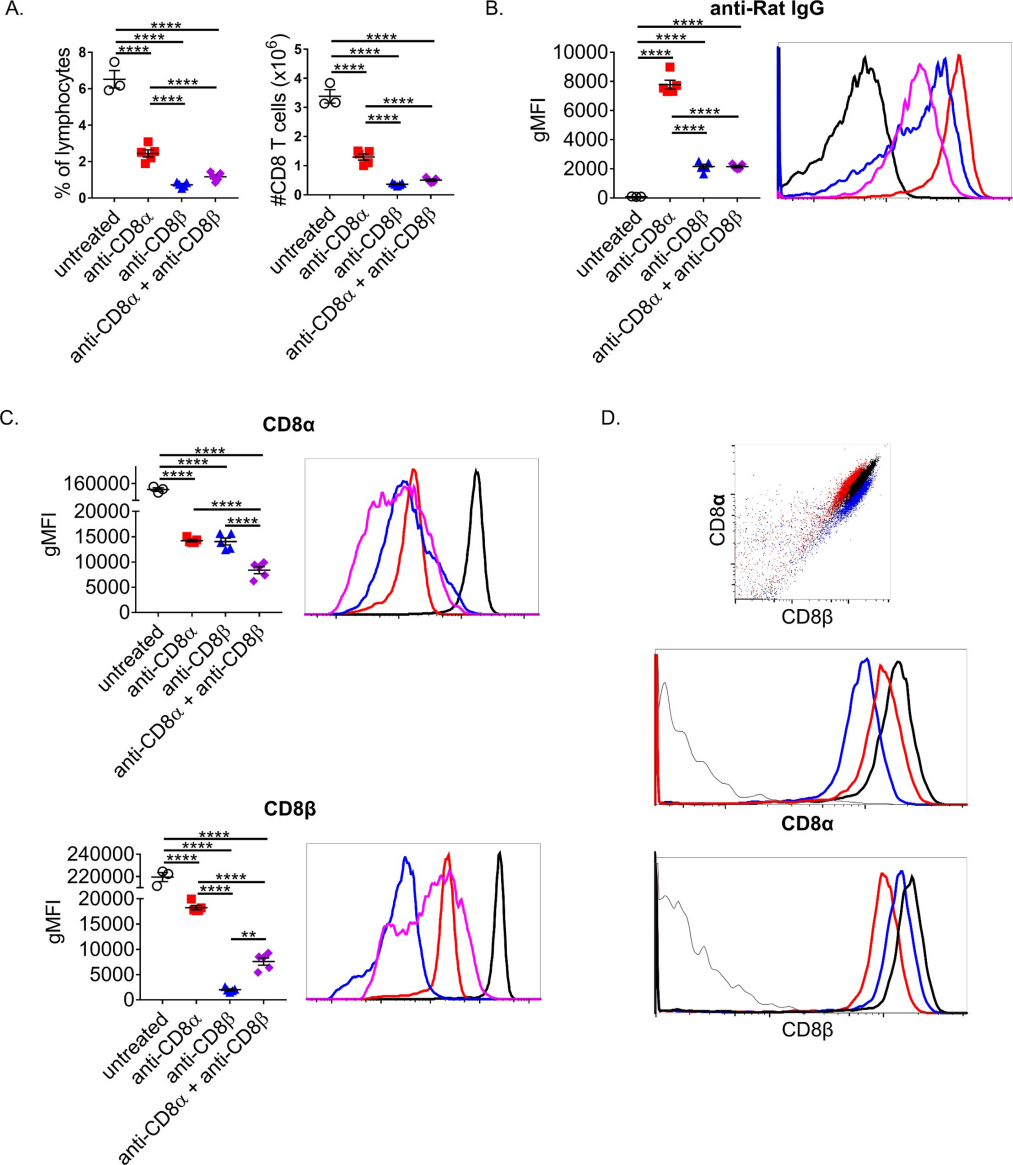
CD8+ T cells are resistant to mAb-mediated depletion and more difficult to identify post-depletion. (A-C) Naïve mice were treated i.p. with a total of 10μg of: anti-CD8α only, anti-CD8β only, or 5μg of both. Splenocytes were isolated and stained the next day. CD8+ T cells were considered CD3+ CD4- B220-. (A) CD8+ T cells as a percentage of lymphocytes and total numbers were calculated. (B) Remaining CD8+ T cell surface bound depleting mAb was determined by staining with Goat anti-Rat IgG. (C) Levels of unbound CD8α and CD8β on surviving CD8+ T cells was determined by staining with the same clones used to deplete. (D) Unmanipulated splenocytes were kept on ice and stained with either anti-CD8α or –β and then stained with the other. Data representative of at least 3 independent experiments. Each point represents the average value of a mouse with 3–5 mice per group.

It is common practice to deplete and verify the efficacy of depletion via flow cytometry with a fluorochrome-labeled mAb of the same clone. Therefore we sought to determine if antibody remains on a target cell after its use for depletion. This is important to assess, as it could interfere with the determination of depletion efficiency and may alter their activation. A low dose of 10μg of either anti-CD8α, anti-CD8β, or 5μg of each together were administered to unmanipulated mice i.p and splenocytes harvested for staining the next day. A low dose of either mAb was able to significantly reduce CD8+ T cells (gated as B220- CD4- CD3+) within the spleen with anti-CD8β having the greatest depletion, similar to what has been previously shown (Fig 1A) [12]. The combination treatment was unable to increase depletion efficiency potentially due to decreased amount of the more effective mAb being used. To test if CD8 was accessible to further staining after depleting mAb treatment, CD8α and CD8β were stained with the same clones used for depletion (Fig 1C). We could detect bound rat IgG on CD8+ T cells of both anti-CD8α and anti-CD8β treated mice but not untreated control treated mice (Fig 1B). A substantial increase in retention of surface bound mAb was noted for anti-CD8α treated over anti-CD8β or combined treated animals. Thus, CD8β engagement likely results in increased internalization of CD8 relative to CD8α engagement. This is further supported by an equivalent drop in CD8α staining when anti-CD8α or –β is used. CD8α was readily detectable in all groups, yet anti-CD8α also resulted in lowered CD8β staining indicating some level of CD8 internalization and/or steric hindrance of anti-CD8α blocking binding of anti-CD8β. To test for steric hindrance, splenocytes were stained with either anti-CD8α or –β alone on ice for 15’ and then the reciprocal anti-CD8 mAb was similarly used to stain (Fig 1D). Surprisingly, both mAbs were able to impede the subsequent staining of the other, showing clearly separate populations when overlaid. The ability of one mAb to block binding of another even when it is targeting a different molecule is concerning as many reports verify their depletion by using a different clone, under the assumption that targeting a different epitope is sufficient. Combining this steric hindrance with mAb-mediated CD8 internalization, the surviving population may be mischaracterized as CD8 negative and remain undetected. Thus, many of these commonly used mAb reagents need to be tested thoroughly for a given system to be sure depletion is sufficient and the ability to verify depletion is adequate.

### CD8+ T cells that survive depleting mAb treatment retain bound anti-CD8α & -β mAb throughout a primary immune response

**Fig 2:**
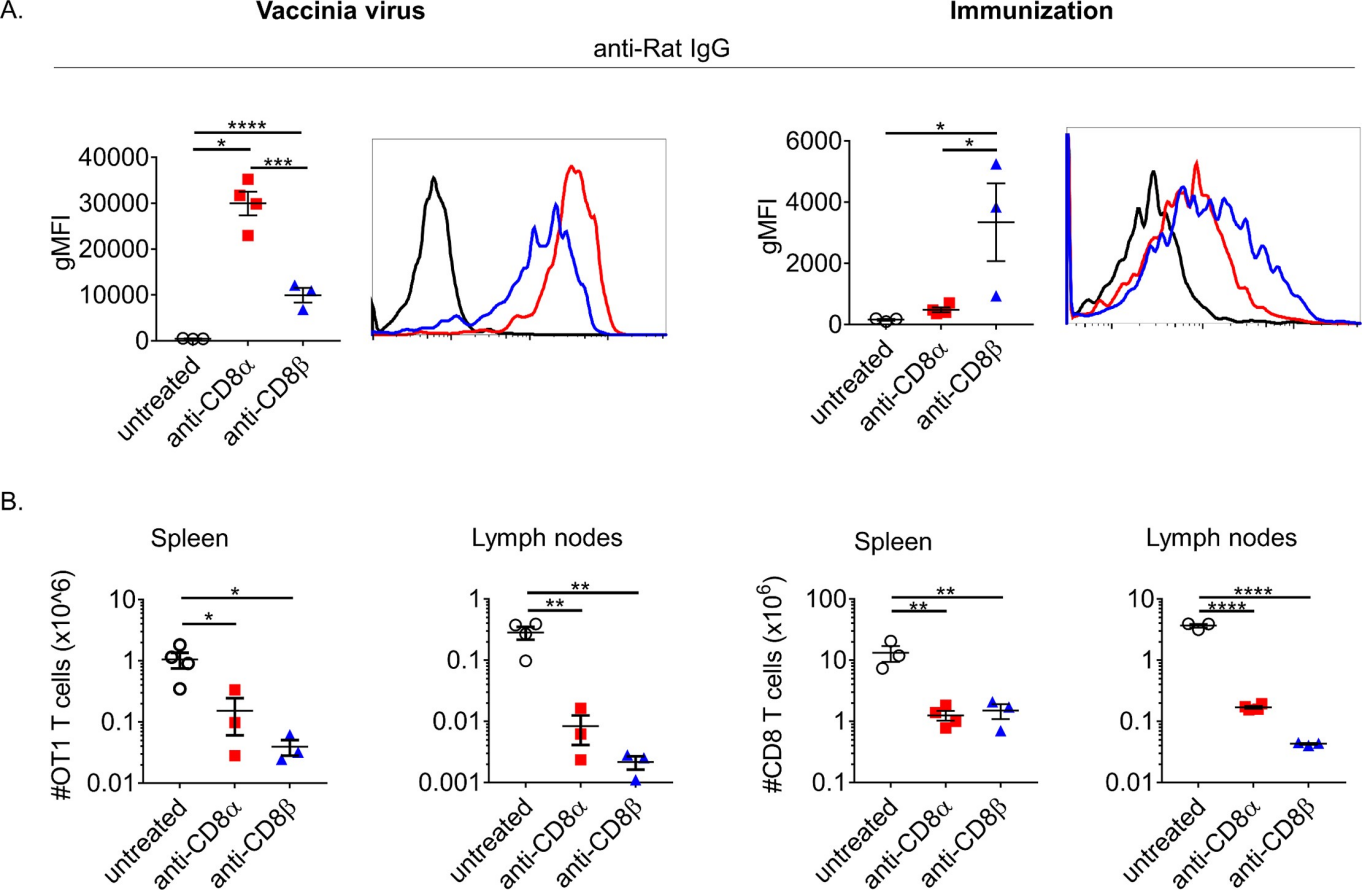
CD8+ T cells that survive depletion participate in immune responses. (A-B) 10^6^ CD45.1 OT1 T cells were transferred i.v. into CD45.2+ mice, treated i.p. with 500μg of anti-CD8α or –β the next day, and immunized or infected. Spleens and lymph nodes were collected on day 5 post-infection or on day 7 post-immunization. CD8+ T cells were considered as CD45.1+ B220-. (A) Surface bound depleting mAb on OT1s was determined by staining with Goat anti-Rat IgG. (B) Remaining OT1 T cells as a percentage of lymphocytes and total numbers were calculated per spleen or pooled lymph nodes. Data representative of at least 3 independent experiments. Each point represents the average value of a mouse with 3–4 mice per group.

To begin to understand how bound depleting mAb could affect an immune response we first sought to address whether mAb remains bound throughout a primary challenge. To address this 10^6^ CD45.1+ OT1 CD8+ T cells (specific for SIINFEKL peptide of ovalbumin) were adoptively transferred into CD45.2+ C57BL/6 mice. Using transferred OT1 T cells ensured a population of antigen-specific CD8+ T cells post-immunization/infection remained after depletion that could be identified with a congenic CD45 marker. The next day mice were treated with a high dose (500μg) of either anti-CD8α or anti-CD8β i.p. or left untreated. For these experiments we chose a high dose of depletion mAb to ensure that the maximum proportion of CD8+ T cells were exposed to a saturating amount of mAb. The following day the depleted mice were either immunized against whole ovalbumin with poly(I:C) and anti-CD40 mAb as adjuvants or infected with vaccinia virus expressing ovalbumin (VV-ova). Seven days after immunization or infection, both anti-CD8α and –β surviving OT1 T cells had readily detectable mAbs bound, confirmed directly by staining for rat IgG (Fig 2A). Interestingly, the levels were different between anti-CD8α and –β dependent on whether the mice were immunized or infected, with immunization responding OT1 T cells retaining more depleting mAb. This may be important in regards to potential direct effects of bound anti-CD8, including signaling and modulation of TCR:peptide-MHC-I interaction that could continually impact these CD8+ T cells and alter their behavior. As expected, both anti-CD8α and –β treated groups had drastically fewer OT1 T cells during the primary challenge to either vaccinia or immunization (Fig 2B). Generally, there were less survivors of the anti-CD8β treatment consistent with the efficiency of depletion prior to challenge (Fig 1A). Although, after immunization, a larger difference in surviving OT1 T cells trended within the LNs compared to the spleen. These results confirmed that bound mAbs are present throughout a response. Furthermore, the differences in total surviving cell number between the spleen and LNs across challenge types suggested both depleting-mAb used and method of challenge may affect T cell localization and their differentiation.

### CD8+ T cells that survive depleting mAb treatment express different differentiation markers

**Fig 3:**
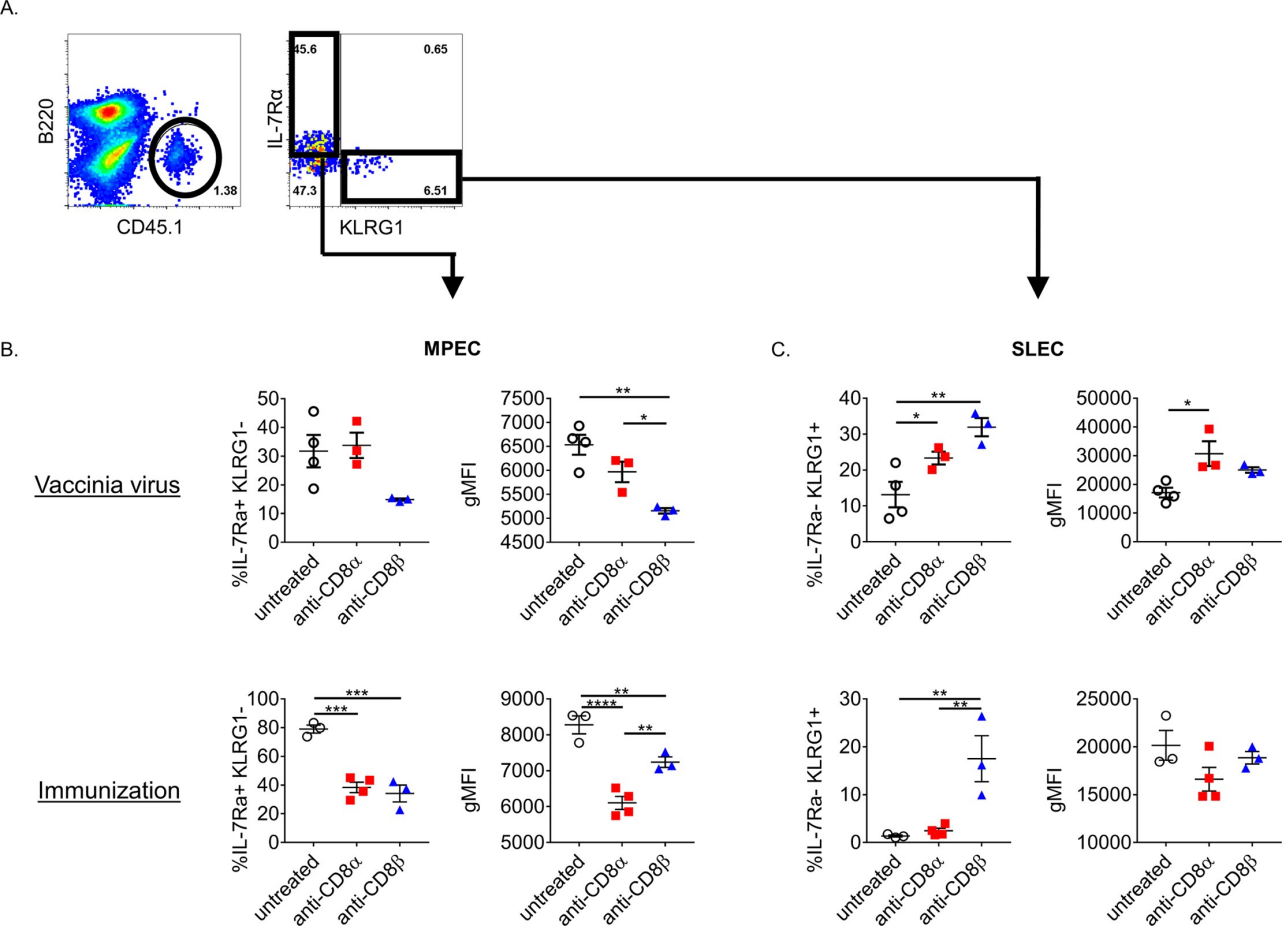
CD8+ T cells that survive depleting mAb treatment acquire distinct differentiation phenotypes. (A) Flow cytometry gating strategy: after gating on singlet and live lymphocytes, B cells were gated away and the CD45.1+ adoptively transferred OT1 T cells were gated on. Subsequently OT1 T cells were assessed for IL-7Rα and KLRG1 expression. (B) Percent of OT1 T cells that are of MPEC phenotype (IL-7Rα+ KLRG1-) and gMFI of IL-7Rα expression of the MPEC cell only. (C) % of OT1 T cells that are SLEC phenotype (IL-7Rα- KLRG1+) and gMFI of KLRG1 of the SLEC cells only. Representative data shown from 3 independent experiments for immunization and 2 experiments for infection. Each point represents the average value of a mouse with 3–4 mice per group.

To begin to determine whether CD8+ T cells that survive mAb-mediated depletion are functionally altered we measured the expression of common differentiation markers at the peak of the response of depletion surviving OT1 T cells. The surface markers IL-7Rα (CD127) and KLRG1, are commonly used to delineate CD8+ T cells that are memory precursor effector cells (MPEC; IL-7Rα^hi^ KLRG1^lo^) and short lived effector cells (SLEC; IL-7Rα^lo^ KLRG1^hi^). Generally, both anti-CD8 mAb treatments resulted in a decreased MPEC phenotype and a commensurate skewing toward an SLEC phenotype. OT1 T cells remaining after treatment with either depleting-mAb with immunization were severely reduced in IL-7Rα expression within the MPEC compartment, as well as by the percentage of MPEC OT1 T cells (Fig 3B). In the context of infection, OT1 T cells treated with anti-CD8β downregulated IL-7Rα similarly, but anti-CD8α treated did not. KLRG1 was upregulated on OT1 T cells surviving either anti-CD8 treatment in immunized or infected mice (Fig 3C). Thus, there is an apparent phenotype imparted directly by the depletion mAb used with some differences noted between types of challenge. To ensure that the isotype of mAbs did not affect the CD8+ T cell response, we also treated mice with IgG1 and IgG2a isotype controls at a high dose prior to immunization and noticed no differences in the surface expression of multiple T cell markers that change with differentiation (S1 Fig). Additionally, it is known that precursor frequency and SLEC phenotype are inversely correlated [13]. Therefore to ensure that differences seen between anti-CD8α and –β surviving CD8+ T cells isn’t due to precursor frequency we compared MPEC and SLEC frequencies after adoptive transfer of a low dose (5x10^4^) or a high dose (10^6^) of OT1 T cells. As to be expected, the ratio of MPEC cells increased with higher transfer, yet the difference between anti-CD8α and –β remained intact demonstrating that the difference in differentiation seen between mAb treatments is not due to precursor frequency (S2A Fig).

### Depletion surviving CD8+ T cells differentially localize

**Fig 4:**
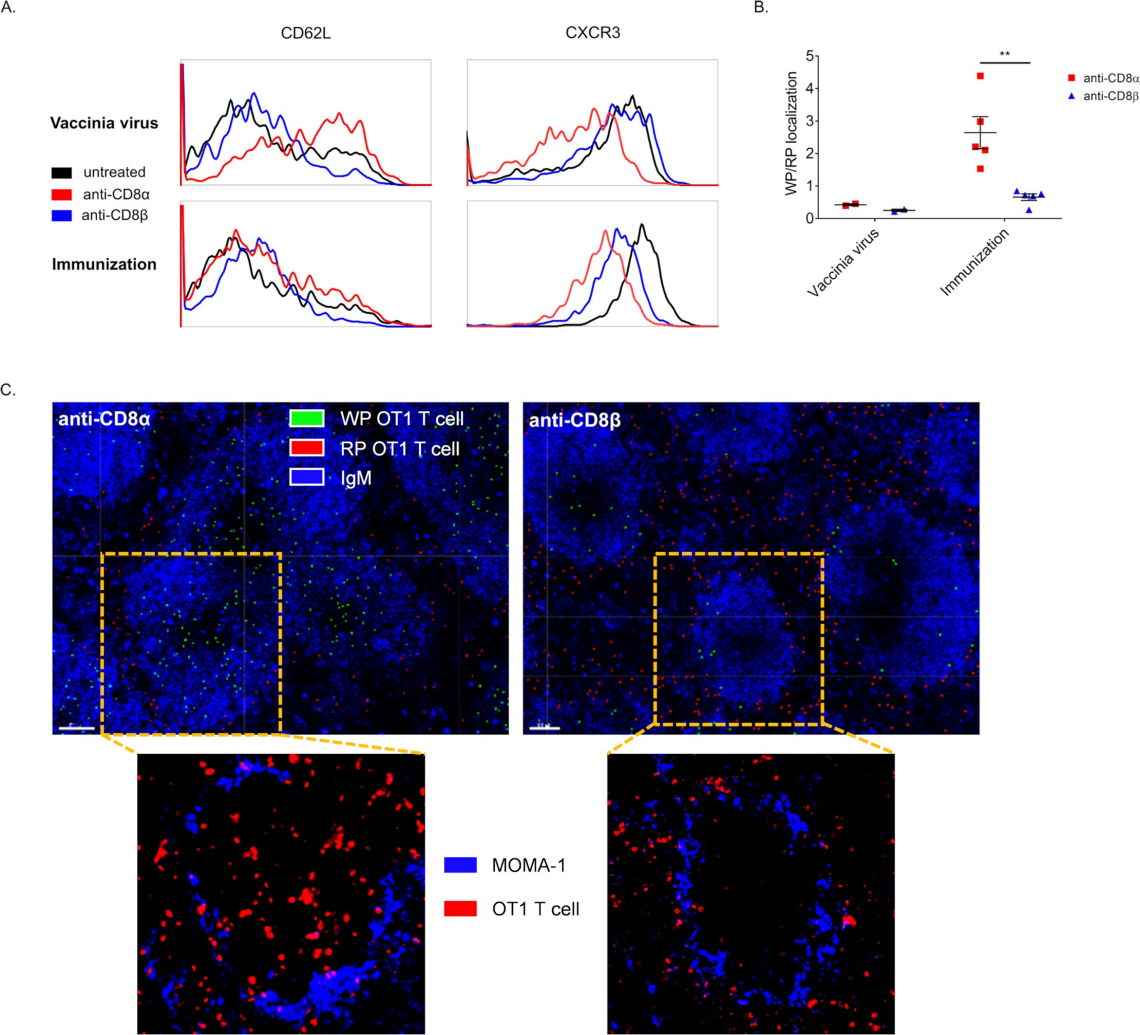
CD8+ T cells surviving depletion differentially localize dependent on mAb used for depletion and type of challenge. (A-B) 10^6^ CD45.1+ OT1 T cells were transferred i.v. into CD45.2+ mice, treated with anti-CD8α or –β the next day, and immunized or VV-ova infected the day after. Spleens were harvested on day 7 post-infection or immunization and either used for flow cytometry or fixed for immunofluorescence imaging. (A) Post-depletion OT1 T cells were stained for levels of CXCR3 and CD62L. (B) CD45.1+ OT1 T cells were quantified as either white pulp (WP) or red pulp (RP) localized and plotted as a ratio. Points represent the average value per mouse determined from at least 3 sections, pooled from 2 experiments. (C) Representative images of spleen sections 7 days after immunization. Top: blue IgM staining is used to highlight the architecture and delineate RP and WP and false color spots were superimposed to show OT1 T cells scored as WP localized (green) and RP localized (red). White bar represents 100μm. Bottom: images magnified to show individual WP regions; blue MOMA-1 (CD169) staining shows metallophilic marginal macrophages and outlines WP regions and red CD45.1 staining depicts CD45.1+ OT1 T cells.

As there were apparent differences in OT1 T cell numbers in spleen and LN between anti-CD8α and –β treated mice, we next stained for select molecules involved in localization that are known to vary in expression across T cell differentiation states 7 days after immunization or infection (Fig 4A). Regardless of type of challenge, the adhesion marker CD62L was significantly higher on OT1 T cells surviving anti-CD8α treatment than both anti-CD8β treated mice and control mice near the peak of the response. The reverse behavior was noted for the inflammatory chemokine receptor CXCR3. These data demonstrated that the mAb used to deplete CD8+ T cells imparts a particular phenotype directly–this is demonstrated starkly by anti-CD8 treatment removing the difference seen in CD62L regulation in hi vs low precursor frequency of OT1 T cells (S2B Fig). The mechanism of this may be similar to what has been seen with use of a non-depleting anti-CD4 mAb, clone YTS177 that activates Rac GTPases and alters trafficking [14]. Further to this point, the non-depleting anti-CD8 mAb, clone YTS105, has also been shown to behave similarly to the aforementioned anti-CD4 mAb where both promote trafficking away from the pancreas and increase numbers of islet-specific T cells in the spleen [15, 16].

To assess if differences in these markers corresponded to differences in localization, we also fixed and froze half of the spleens used above for immuno-fluorescence imaging. On imaged splenic sections, the depletion-surviving OT1 T cells were identified by congenic CD45.1 staining and quantified using Imaris software for white pulp (WP) and red pulp (RP) localization, where CD45.1+ OT1 T cells are depicted with superimposed dots and green if scored as WP localized (delineated by MOMA-1 and IgM staining) and red if scored as RP localized (outside of areas circumscribed by MOMA-1 and IgM) with (Fig 4C). Unaltered images of single WP areas, with higher magnification, are shown below (Fig 4C). Strikingly, there was more than a 3-fold increase in white pulp localized anti-CD8α surviving OT1 T cells after immunization relative to anti-CD8β survivors, which mimicked either mAb treatment group after VV-ova infection (Fig 4B and 4C). Both anti-CD8 groups in infection and anti-CD8β with immunization show similar localization to what has been reported previously for the primary response of endogenous CD8+ T cells against *Listeria monocytogenes* and the response of gBT-transgenic T cells against lymphocytic choriomeningitis virus [17, 18]. Given this data, we conclude that both the mAb used to deplete CD8+ T cells as well as the type of challenge impart a particular phenotype to surviving T cells that can result in a difference in localization.

### CD8+ T cells that survive depleting mAb treatment have different cytotoxic capabilities dependent on the depleting-mAb used

**Fig 5:**
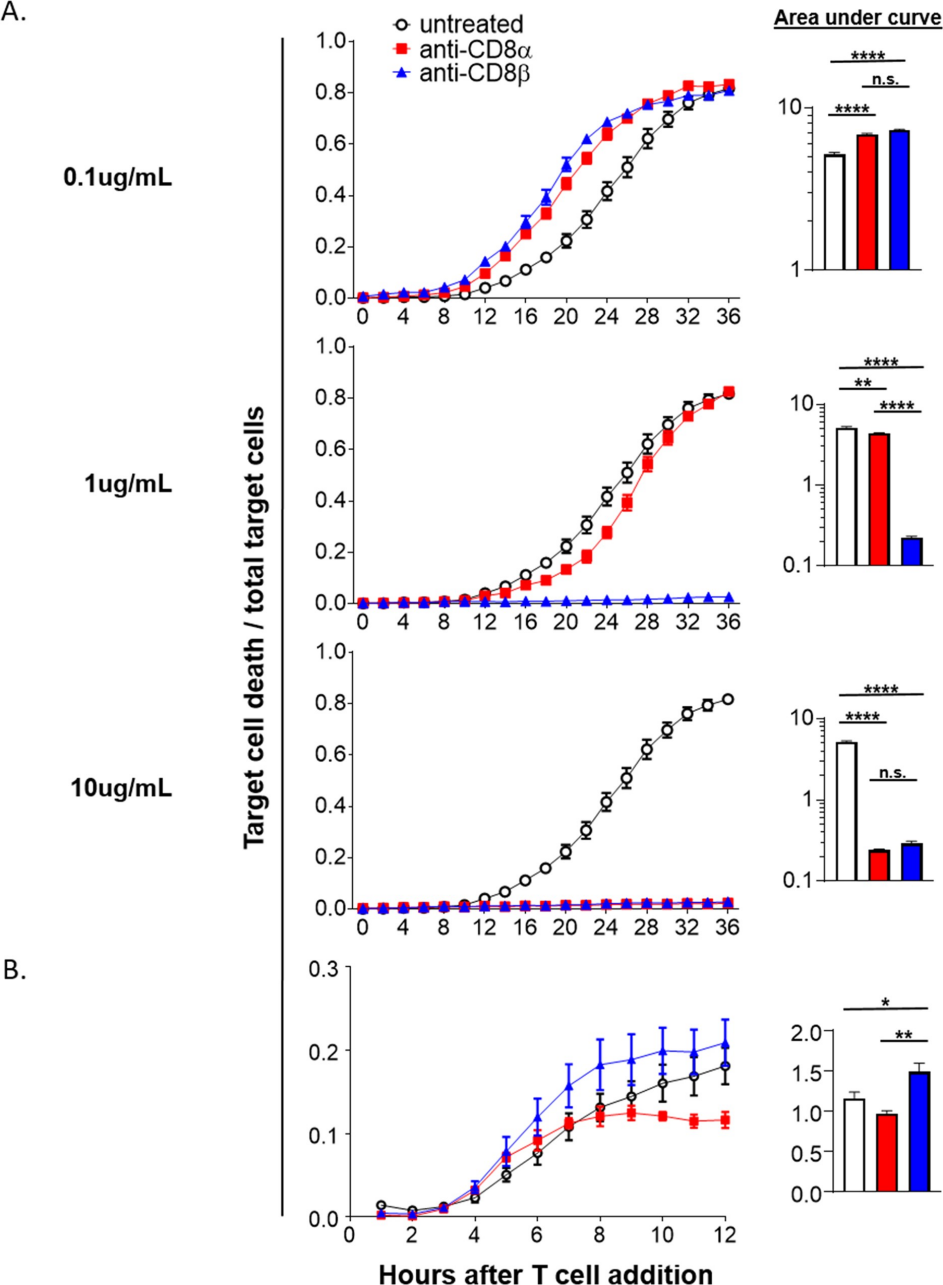
Anti-CD8 mAbs have direct effects on target recognition and indirect effects on cytotoxic function. (A-B) IncuCyte cytotoxicity assays presented as the ratio of target cell death/total target cells over time. Activated OT1 T cells were added to previously plated B16.OVA.RFP tumor target cells. (A) *in vitro* SIINFEKL activated OT1 T cells were plated at an effector to target ratio of 10:1. Anti-CD8 mAbs were then added at multiple concentrations with at least 4 technical replicates per condition. Data is representative of 3 independent experiments. (B) OT1 T cells were adoptively transferred into naïve mice that were anti-CD8 treated the next day. The following day the mice were immunized and the OT1 T cells FACS isolated from pooled lymph nodes and spleens 7 days later. OT1 T cells were then plated at an effector to target ratio of 1:1 with at least 3 technical replicates per treatment group. Data is representative of 2 independent experiments.

Due to differences in CD8+ T cell phenotype during activation after anti-CD8 treatment, it was necessary to address whether these translated into differences in function. Two questions were immediately evident: i) do the depleting-mAbs perturb cytotoxic function directly, meaning do they affect CD8 T cell function purely by virtue of them being within a system with target cells and ii) are the surviving CD8+ T cells efficient cytotoxic effectors given that their differentiation is altered by bound anti-CD8 mAbs during stimulation? To address the first question OT1 splenocytes were stimulated with SIINFEKL peptide *in vitro* then assayed for cytotoxicity on a per cell basis using an IncuCyte imaging system. Briefly, OT1 T cells were mixed with previously plated OVA expressing B16 melanoma target cells at a known ratio and anti-CD8 mAbs were added at various concentrations along with an active caspase-3/7 fluorescent dye. These B16.OVA target cells also express red fluorescent protein (RFP) allowing for easy discrimination from added CD8+ T cells and therefore dying B16 cells were quantified as RFP+ caspase-3/7+ at each time point. We predicted that we would see opposing effects of the mAbs because previous reports demonstrated that the anti-CD8α mAb used in these studies keeps CD8 in a conformation with higher affinity for MHC-I affording increased MHC-I multimer staining [19–21]. In contrast, the anti-CD8β been shown to substantially inhibit the binding of TCR to peptide-MHC-I [20]. To our surprise, both anti-CD8α and –β increased cytotoxicity at the lowest dose tested, 0.1μg/mL (Fig 5A). In contrast, with increased doses we saw a stark stunting of target cell death when anti-CD8β was added. Anti-CD8α showed a clearer dose-dependent inhibition of killing. At 1μg/mL only modest inhibition of killing was evident whereas at 10ug/mL the inhibition matched that of anti-CD8β. From this we conclude that at a relatively low dose anti-CD8β effectively blocks TCR recognition of peptide:MHC-I and the stabilizing effect anti-CD8α has on TCR:MHC-I multimer staining is not enough to effect cytotoxicity at high doses. At a high dose both mAbs effectively inhibit cytotoxicity indicative of direct signaling downstream of CD8 engagement being inhibitory.

Knowing that CD8+ T cells surviving depletion can have differing phenotypes, we addressed our second question of whether CD8+ T cells activated in the presence of anti-CD8 *in vivo* had altered cytotoxic function. For this, OT1 T cells were adoptively transferred, treated with either anti-CD8 mAb or left untreated, immunized the next day, and 6–7 days later purified by FACS or magnetic-bead isolation. The same IncuCyte cytotoxicity assay was run as before without addition of anti-CD8 mAbs to the media. Consistent with our phenotypic data suggesting particularly strong effector differentiation, anti-CD8β surviving OT1 T cells after immunization also had increased target killing (Fig 5B). This was in contrast to anti-CD8α surviving OT1 T cells that have a decrease in cytotoxicity compared to untreated OT1 T cells. It is important to note that the cytotoxicity of CD8+ T cells generated from our subunit vaccination are heavily skewed toward a central-memory phenotype (Fig 3, compare the control cells of infection vs. immunization) and therefore were not expected to show the same cytotoxicity curve or scaling of the ratio of dying target cells to total target cells [22–27]. Together with our data on the effect of anti-CD8 present only at the time of the assay, we conclude that these anti-CD8 mAbs can both positively and negatively regulate cytotoxic function. At high concentrations both anti-CD8 mAbs negatively affect cytotoxicity. For anti-CD8β, this is likely accomplished by direct interference of the TCR:peptide-MHC-I interaction. For anti-CD8α, inhibition may also be derived from CD8 signaling that negatively affects the cell. This is supported by low concentration enabling enhanced effector function that turns to decreased function at higher doses over a much larger range compared to anti-CD8β treatment. However, at the lowest dose, both mAbs are likely causing CD8 signaling as they both increase effector function when delivered during the assay only.

### CD8+ T cells that survive anti-CD8α or -β mAb treatment form memory T cells with differing phenotypes

As part of the adaptive immune system, CD8+ T cells are integral to how organisms are able to respond to pathogens with increased robustness on subsequent encounters. This ‘memory’ is due to CD4, CD8, or B lymphocytes that, after clearance of a pathogen/antigen and subsequent contraction of the effector population, leave behind a small population of cells that undergo differentiation into long-lived lymphocytes able to respond again with increased kinetics and effector potential. CD8+ T cells can form several subsets of memory T cells that have different functionality and phenotypes. We therefore asked whether the CD8+ T cells that survive anti-CD8 mAb treatment form a memory T cell population and whether those memory T cells have differing phenotypes dependent on the anti-CD8 mAb used.

**Fig 6:**
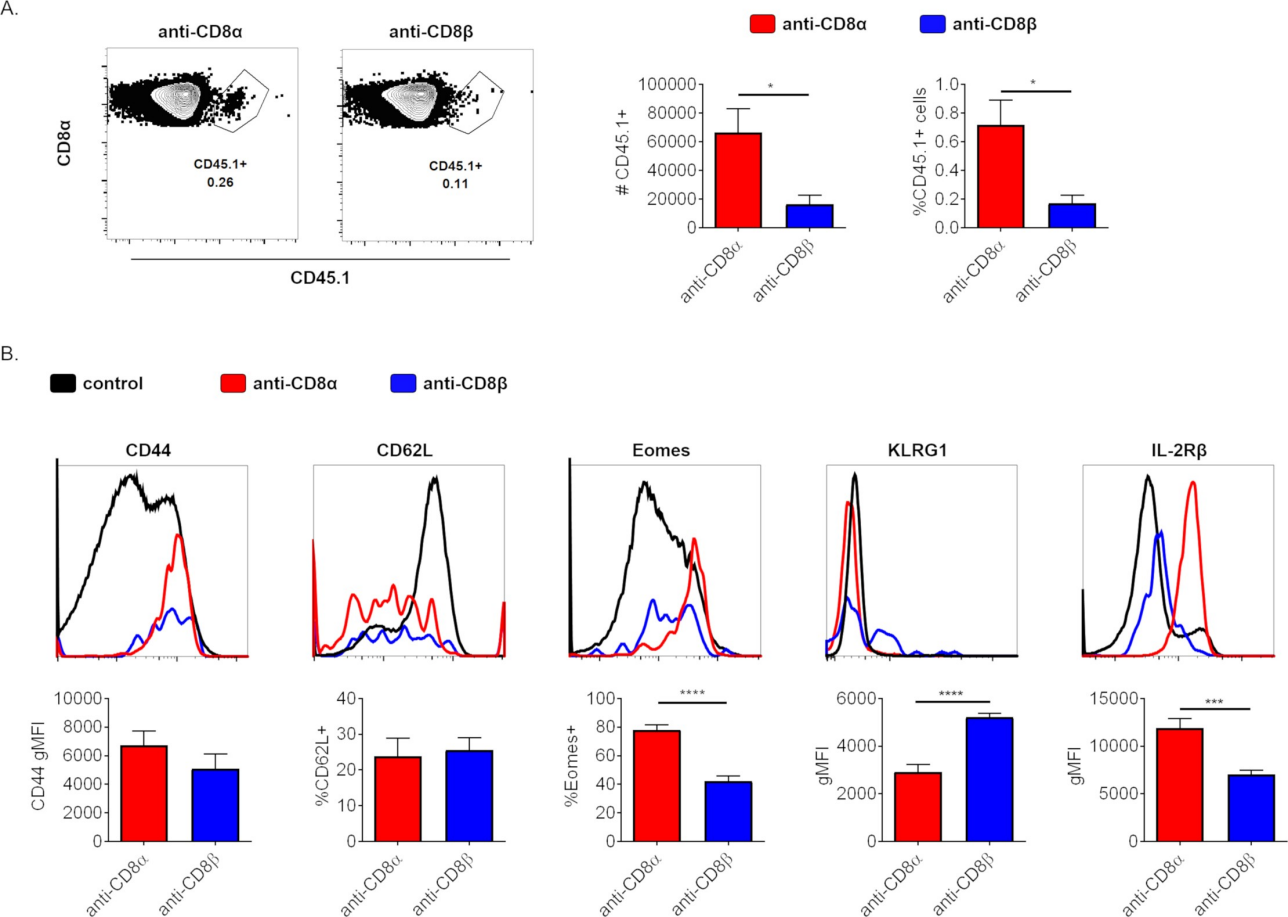
CD8+ T cells that survive anti-CD8α or -β mAb treatment form memory T cells with differing phenotypes. (A-B) 10^6^ CD45.1 OT1 T cells were transferred i.v. into CD45.2+ mice, treated with anti-CD8α or –β the next day, and immunized the day after. Mice were allowed to rest, unmanipulated for 62–67 days before staining of splenocytes by flow cytometry. (A) Representative flow plots of memory CD45.1+ OT1 T cells surviving treatment with depleting anti-CD8α or -β mAb with quantification in total numbers and as a percent of CD8+ B220- cells. (B) Comparison of select markers associated with memory and differentiation of cells depicted in A. Black control line in histograms depicts CD8+ B220- T cell population as a whole for reference, except for CD44 where black line depicts B220- cells. Data are representative of 2 independent experiments pooled with 6 mice total.

Generally, after the primary response and subsequent contraction of responding lymphocyte populations, CD8+ T cells form central memory T cells that have high proliferative potential and reside within SLOs as well as effector memory T cells that retain cytotoxic/inflammatory functionality and recirculate between peripheral tissues and SLOs [28] We predicted that CD8+ T cells surviving anti-CD8α treatment would form memory T cells that are skewed toward central memory, whereas anti-CD8β T cells would be skewed toward effector memory in line with their appearance and behavior during the primary response. To test this CD45.1+ OT1 T cells were transferred into congenic mice, treated with anti-CD8α or -β the next day, and subsequently immunized another day later as before. To confirm that mice treated with either anti-CD8α or -β and subsequently immunized formed a memory T cell population, splenocytes were harvested for analysis by flow cytometry 62–65 days later, ensuring a long-lived memory T cell population. We first noted that the ratio of remaining anti-CD8α or -β T cells recapitulated the ratio seen during the primary response, with significantly more CD8+ T cells remaining in anti-CD8α treated mice (Fig 6B and Fig 2). Equivalent, high expression of the activation marker CD44 and similar expression of the lymph node homing receptor CD62L supported that both depleting mAb-surviving populations were indeed memory T cells (Fig 6B). We then asked whether those memory T cells derived from either depleting mAb treatment survivors were different from each other by assessing for cell surface markers as well as the transcription factor Eomesodermin (Eomes) that is associated with memory T cells. [29–31]. Relative to anti-CD8β surviving CD8+ T cells, anti-CD8α surviving memory CD8+ T cells have increased Eomes expression, a transcription factor shown to drive the central memory phenotype in particular (Fig 6B) [32]. Consistent with this, anti-CD8α surviving CD8+ T cells also had increased IL-2Rβ (CD122) expression, a gene whose expression is directly promoted by Eomes and is part of the IL-15 receptor whose signaling supports memory CD8+ T cell differentiation and maintenance (Fig 6B) [31, 33]. In contrast, anti-CD8β surviving, memory CD8+ T cells displayed a more effector memory-like phenotype. The NK and effector T cell marker, KLRG1, is increased in anti-CD8β surviving memory T cells as a whole compared to those surviving anti-CD8α [34, 35]. These differences suggested a further potential discrepancy in the protective capability of memory T cells that survived anti-CD8α or -β mAb-mediated depletion.

To assess whether there is a difference in protection between anti-CD8α or -β surviving, memory CD8+ T cells, mice harboring either anti-CD8α or -β surviving memory T cells were secondarily challenged with VV-OVA and assessed for control of viral PFUs within the ovaries 4 days after infection. Despite significantly increased numbers of memory T cells after anti-CD8α treatment compared to anti-CD8β, there was no difference detected in control of viral burden (S3 Fig). Therefore, memory CD8+ T cells surviving anti-CD8β treatment have increased cytotoxic functionality on a per cell basis, which is consistent with anti-CD8β surviving CD8+ T cells during the primary response (Fig 5). This further supports anti-CD8β surviving CD8+ T cells being largely effector memory in nature.

### Anti-CD8 mAbs can alter the metabolism of activating CD8+ T cells

**Fig 7:**
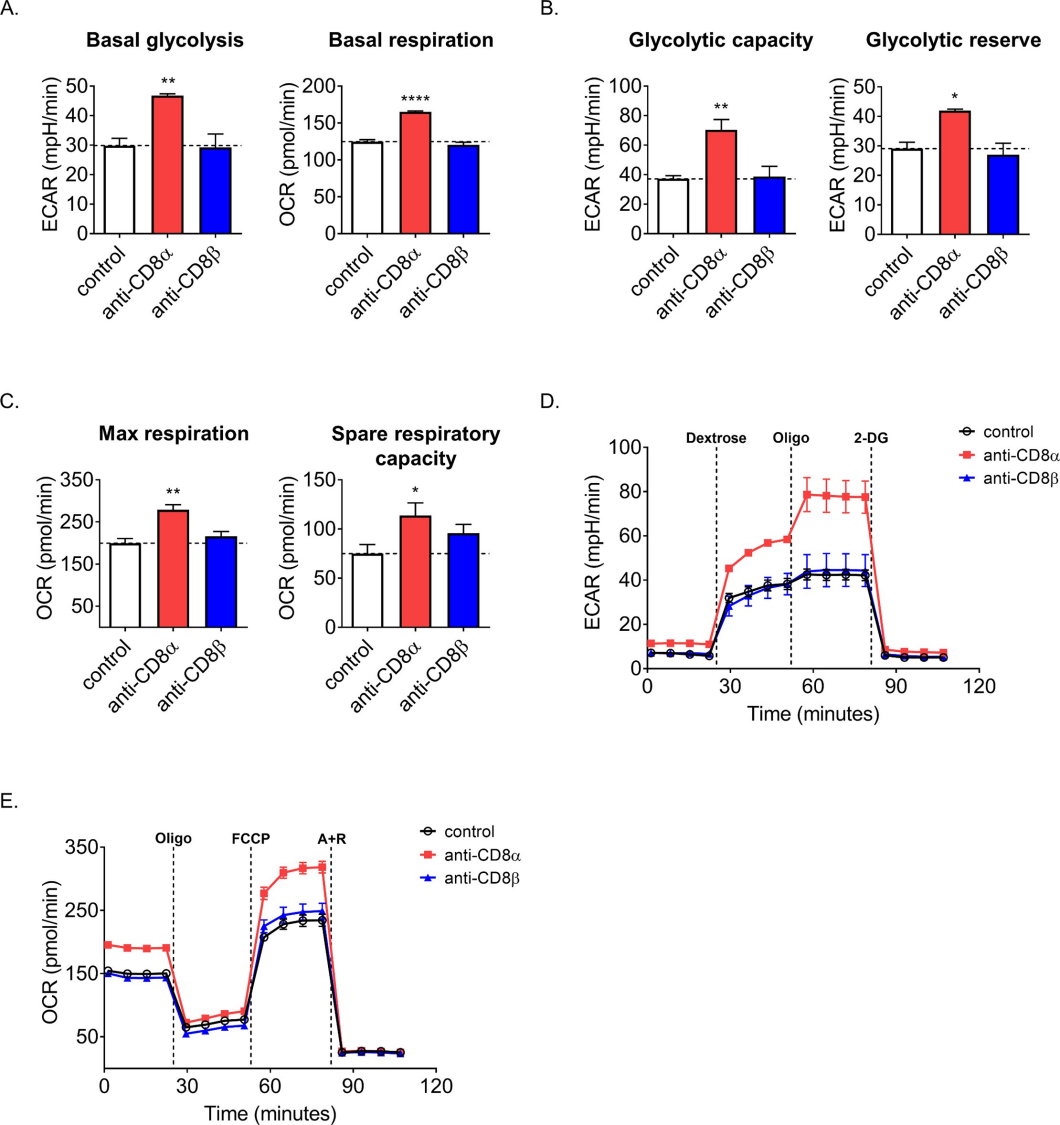
Anti-CD8α boosts the metabolism of CD8+ T cells during activation. (A-D) CD8+ T cells were isolated from unmanipulated mice and anti-CD3ε /anti-CD28 stimulated for 2–3 days in the presence of either anti-CD8α or –β prior to metabolic flux profiling. Dashed line represents control value. (A) Resting ECAR and OCR of blasting CD8+ T cells. (B) Maximum glycolysis was obtained by injection of oligomycin and glycolytic reserve was then calculated by subtracting basal ECAR from the maximum. (C) Maximum respiration was obtained by injection of FCCP and spare capacity was then calculated by subtracting basal OCR from the maximum. (D) Plot of ECAR and (E) OCR measurements overtime with drug injections indicated. Data are representative of 3 experiments performed with at least 3 technical replicates per treatment group.

Recently there has been a growing understanding and appreciation of how intertwined metabolism is to T cell function and fate. Increased utilization of OXPHOS and the tricarboxcylic acid (TCA) cycle, partially dependent on fatty acid oxidation, is critical for the development and maintenance of memory T cells [36]. However, effector T cells rely heavily on aerobic glycolysis (Warburg metabolism)–producing lactic acid from glucose [37]. Some therapies are already exerting metabolic control over immunity. As an example, mAbs that target CTLA4 and PD1 or its ligands, PD-L1/2, have become potent weapons in the armamentarium against cancer by removing an impediment to sustained anti-tumor T cell activity [38]. Part of the efficacy of anti-PD1 likely derives from its ability to block metabolic regulation by PD1 of both glycolysis and OXPHOS [39]. This is because T cells involved in chronic infectious or tumor responses often adopt an exhausted phenotype, a state characterized by relative functional and metabolic quiescence [40]. Knowing that other immunomodulatory therapies have demonstrated influence on CD8+ T cell metabolism and that CD8 is the co-receptor for the T cell receptor (TCR) and therefore influences activation, we predicted that use of anti-CD8 mAbs may alter the metabolism of CD8+ T cells as well. We decided to assess metabolic differences after 2–3 days of stimulation, when rapid proliferation is occurring, and therefore would likely have the greatest energy demand. As an added benefit, introducing anti-CD8 mAbs *in vitro* at the time of stimulation would mimic how these mAbs would affect CD8+ T cells *in vivo* as they are typically used to deplete CD8+ T cells prior to experimentation and potential activation. To test how mAbs would affect CD8+ T cells, spleen and lymph nodes were pooled and CD8+ T cells were magnetically enriched by negative selection and seeded onto an anti-CD3ε coated plate in media with anti-CD28. Either anti-CD8α or –β were added or no anti-CD8 was added as a control. Using a Seahorse XFe96 metabolic flux analyzer, we found that anti-CD8α treatment was uniquely able to increase multiple metabolic measures, including basal oxygen consumption rate (OCR, representing mitochondrial respiration) and extracellular acidification rate (ECAR, a measure of lactic acid production from aerobic glycolysis) relative to untreated, stimulated control cells (Fig 7A). To look more fully into the glycolytic potential, oligomycin was injected to inhibit ATP-synthase and thus force all energy to come from lactate fermentation, establishing the maximum glycolytic capacity (Fig 7B). The glycolytic reserve (difference between maximal and basal glycolysis) was also found to be uniquely improved with anti-CD8α mAb treatment (Fig 7B). From these glycolytic measures any source of background pH increase was determined by injecting 2-deoxyglucose (2-DG), a competitive inhibitor of glycolysis, and subtracted (Fig 7D). Similarly, injection of carbonyl cyanide p-trifluoromethoxyphenylhydrazone (FCCP) to uncouple the mitochondrial membranes afforded the maximal respiratory rate (Fig 7C). Spare respiratory capacity was determined as the difference between maximal and basal respiration and similarly increased with anti-CD8α (Fig 7C). From these OXPHOS measures, background O2 consumption was determined by injection of the electron transport chain inhibitors antimycin A & rotenone and subtracted (Fig 7E). In all of these measures anti-CD8α was able to increase metabolism compared to control, whereas anti-CD8β had no effect. Importantly, the anti-CD8α-driven effect seen and anti-CD8β’s failure to have an effect is not due to promotion or blockade of TCR:peptide-MHC-I interaction respectively, as these assays were performed with anti-CD3 mAb-mediated stimulation. This suggested that mAbs commonly used to deplete cell types may leave behind a small, yet potentially important population of surviving cells that may behave differently than expected and therefore affect results in different ways. Additionally, simply cross-linking CD8 is likely not the source of the increased metabolism seen with anti-CD8α as anti-CD8β would be expected to create a similar effect. These results may be more generalizable as similar behaviors and binding from the clones used here and non-depleting clones may mean that these effects can be achieved without removal of most of the CD8+ T cell population.

## Discussion

Despite the decades of work done with lymphocyte depleting mAbs, there is a surprising dearth of information as to how these reagents work and what unintended side effects occur from their use. Herein, we demonstrate that CD8+ T cells that survive mAb-mediated depletion can retain the depleting mAb on their surface, alter the surface expression of CD8, and are able to participate in immune responses. Surviving CD8+ T cells display phenotypic and functional differences dependent on the antibody used to deplete them as well as the context in which they are activated. Surviving CD8+ T cells from mice treated with anti-CD8β are more SLEC-like and have increased cytotoxicity relative to untreated mice. In contrast, CD8+ T cells surviving anti-CD8α treatment are unable to completely differentiate and strikingly, in the case of immunization, these CD8+ T cells are less able to emigrate from the white pulp of the spleen. These differences are reflected in the memory T cells that develop, whereby anti-CD8α surviving T cells are higher in Eomes and IL-2Rβ whereas anti-CD8β surviving memory T cells have higher relative KLRG1, suggestive of a more central or effector memory-like population respectively. It is unclear the mechanism(s) involved in such differential phenotypes and behaviors, but part of the explanation likely comes from anti-CD8α treatment uniquely increasing metabolism during activation. This highlights the importance of knowing how well depleting reagents and protocols used within a given experimental system work and that it may be worthwhile to track the surviving population throughout the experiment.

As shown herein, how the remaining CD8+ T cell population appears after depletion is dependent on several parameters. The clone of depleting mAb used can substantially differ in its effect on CD8 internalization as well as interfere with subsequent staining of CD8, both of which can prevent remaining CD8+ T cells from being accounted for. Overly conservative flow cytometric gating can fail to identify CD8+ T cells staining much lower than in unmanipulated animals. Reduced staining is most notable with the anti-CD8β used here, as CD8 is internalized quite substantially. Furthermore, it may be particularly difficult to detect depletion-surviving CD8+ T cells participating in a response as CD8 and CD3 can be downregulated with stimulation. In such a scenario it may be informative to ensure that numbers are not increasing in the “negative” population.

For the purpose of depleting CD8+ T cells as completely as possible the anti-CD8β used here (clone 53–5.8) was significantly more effective than the anti-CD8α (clone 53–6.7). In an attempt to increase depletion efficiency, we combined a low dose treatment of either mAb to target both CD8 subunits, however, this strategy did not result in increased depletion efficiency relative to anti-CD8β alone. The fact that more CD8+ T cells survive dual treatment than anti-CD8β alone is likely simply due to there being less anti-CD8β in circulation relative to anti-CD8β treatment alone. Unfortunately, use of a secondary mAb against the depleting mAb did not increase depletion effectiveness substantially and comes with its own concerns [41]. Thus, while we cannot recommend the best depletion strategy, we can say that use of 53–6.7 is contraindicated both due to its reduced efficacy of depletion and because the cells that survive are more likely to form a homogenous population of central memory T cells that could complicate longer term studies. However, use of anti-CD8β has its own caveat in that the CD8+ T cells that survive depletion are likely to have increased cytotoxic function and therefore may be more effectual relative to anti-CD8α surviving CD8+ T cells in certain circumstances, particularly during the primary response.

For clinical applications, the use of depletion antibodies is a double edged sword. Depletion of lymphocytes is done to prevent transplant rejection or subdue autoimmunity, yet this sets the stage for robust homeostatic proliferation of the surviving clones creating a large effector-memory pool that is resistant to co-stimulation blockade and can lead to transplant rejection. Indeed, depleting mAbs used clinically, such as anti-CD3 and anti-CD52, target multiple lymphocyte populations creating profound immunosuppression [42, 43]. As no depletion can be complete, it may be worthwhile to test the use of non-depleting mAbs clinically as the issue of homeostatic proliferation is averted and presumably their use affords less immunosuppression. To combat autoimmunity, non-depleting mAbs have shown progress in type-I diabetes and arthritis [15, 16, 44, 45], but for depleting mAbs it may be worthwhile to more specifically target cell subsets. In this regard, anti-Gr1 therapy could hold promise by depleting memory CD8+ T cells only [3] and sparing naïve T cells, which by definition are not autoreactive. Of course, anti-Gr1 therapy would deplete neutrophils and some monocytes, but these cell types are rapidly replaced and so immunodeficiency caused by their absence would likely be limited. Interestingly, anti-CD8 mAbs have demonstrated utility with human cells *in vitro* for altering the threshold for activation, effectively blocking autoimmune cell stimulation while preserving pathogen-specific responses [5]–the mechanism of which is impeding TCR:peptide-MHC-I interaction, similar to the anti-CD8β used here. Besides use of mAbs as a tool to remove cell types, the use of mAbs as an agonist or antagonist of immune system molecules has become a research priority and has demonstrated immense promise.

We believe that the unique behavior of CD8+ T cells surviving anti-CD8α versus anti-CD8β treatment is due to combinatorial effects of perturbed TCR:peptide-MHC-I interaction and direct anti-CD8 induced CD8 signaling. However, an alternative hypothesis is that particular subsets of CD8+ T cells are more resistant to certain mAbs and therefore their use selects for different populations. This would potentially be due to either some resistance to complement mediated lysis, phagocytosis by macrophages, cytotoxicity from NK cells, or a combination thereof. The mAbs used here are rat IgG2a (53–6.7) and IgG1 (53–5.8). Rat IgG2a and IgG1 sequences are very similar to each other and most homologous to mouse IgG1 suggesting they both primarily bind the activating murine FcγRIII [46]. Together with studies that have tested the mechanisms of mAb-mediated lymphocyte depletion in homeostatic conditions and during persistent infection, it is apparent that monocytes and macrophages are the main effector cells and complement has little to no effect [47–49]. Indeed, neither 53–6.7 nor 53–5.8 bind complement well [12]. Therefore, given the similarity in how these mAbs mediate killing, it would be unlikely that these mAbs preferentially select for a different surviving population.

Interest in increasing the cytotoxicity and functionality of T cells using mAbs has led to effective therapies, generally targeting inhibitory receptors. Immunomodulation, i.e. increasing the perceived TCR signal, could be another way to achieve similar robust T cell responses. The co-receptors, CD4 and CD8, are thought to primarily support TCR/CD3 signaling by acting as a conduit for Lck, a Src family kinase that phosphorylates the immunoreceptor tyrosine-based phosphorylation motifs (ITAMs) on TCR/CD3ζ-chains, that is bound to the co-receptor’s cytoplasmic tail and is brought into proximity of the TCR by CD8:MHC-I interaction. It is estimated that co-receptor bound Lck is 10 times more likely to interact with TCR/CD3 than freely diffusing Lck [50]. However, it has also been demonstrated that CD8 can subtly increase the TCR/CD3 signaling by binding to MHC-I directly [50–52]. As the increase in TCR/CD3 signaling by virtue of CD8:MHC-I interaction is thought to be small, yet important, it makes sense that any significant increase in this interaction could have substantial effects. Recently, it was demonstrated that a Fab fragment from an anti-CD3ε agonist mAb could be used to potentiate TCR:peptide-MHC-I signaling [4]. Similarly, it has been shown that the anti-CD8α used here (clone 53–6.7) holds CD8 in a conformation with a higher affinity for MHC-I [21]. Importantly, this change in conformation holds true when 53–6.7 is made into a Fab fragment, potentially allowing it to behave similar to the anti-CD3ε Fab and increase activation of only T cells interacting with cognate peptide-MHC-I. The combinatorial use of anti-CD3ε Fab and anti-CD8α may well prove synergistic and allow for more potent increases in TCR signaling from weakly agonistic peptide-MHC-I combinations. This could be useful for stimulating CD8+ T cell killing of tumor cells, generally thought to be lower affinity targets. It has been shown that tumor infiltrating T cells have an intrinsic metabolic defect as part of the exhausted phenotype, therefore an added benefit of anti-CD8 mAb use may be the boosting of metabolism, in particular respiration that could increase memory differentiation and persistence to counteract exhaustion in CD8+ T cells [39, 53–55]. PD1 has been shown to regulate the activity of Peroxisome proliferator-activated receptor-gamma coactivator (PGC)-1α a transcriptional coactivator, thereby decreasing the transcription of glycolytic and respiratory machinery. The combinatorial use of anti-CD3ε Fab and anti-PD1 has been shown to further increase the control of tumor metastases over the use of anti-CD3ε alone [4, 56]. Both the potential to modulate TCR:peptide-MHC-I interaction by virtue of where/how a mAb binds to CD8 and the ability of CD8 to act as a signaling molecule make it an attractive target for immunomodulatory therapies.

In conclusion, we have shown that CD8+ T cells can have altered phenotype and function dependent on both the mAb used to deplete them and the conditions in which they are activated, making predictions about how they will behave in a particular experimental situation difficult. Our results support literature showing that anti-CD8 mAbs can be used to affect cytotoxic and trafficking functions of CD8+ T cells. Further, we demonstrate that part of these mAb-derived effects could be due to shifts in the metabolic profile of the CD8+ T cells themselves. These results highlight the potential use of these, or similar mAbs, have for immunomodulatory therapies to ameliorate autoimmunity as well as promote tumor immunity.

## Supporting information

S1 Fig Antibody isotype does not alter the CD8+ T cell response. 10^6^ CD45.1+ OT1 T cells were transferred i.v. into CD45.2+ C57BL/6 mice and the next day a high dose (500μg) of isotype control antibody was administered i.p. The mice were immunized the next day and splenocytes harvested 7 days later.
(TIF)

S2 Fig Precursor frequency is not responsible for phenotypic differences between anti-CD8α and –β treated CD8+ T cells. (A-B) Either 5x10^4^ or 10^6^ CD45.1+ OT1 T cells were transferred i.v. into CD45.2+ C57BL/6 mice and the next day a high dose (500μg) of anti-CD8α or –β was administered or no mAb given as a comparison. The mice were immunized the next day and splenocytes harvested 7 days later. (A) The percentage of OT1 T cells that are MPEC (IL-7Rα+ KLRG1-) or SLEC (IL-7Rα- KLRG1+) phenotype. (B) The percentage of OT1 T cells that have downregulated CD62L. Note that anti-CD8 mAb treatment perturbs normal difference seen between the low vs hi precursor frequency.
(TIF)

S3 Fig Protective capacity of memory CD8+ T cells that have survived anti-CD8α or -β differ. 10^6^ CD45.1+ OT1 T cells were transferred i.v. into CD45.2+ C57BL/6 mice and the next day a high dose (500μg) of either anti-CD8α or -β was administered i.p. The mice were immunized the next day and allowed to rest for 62 days before infection with 10^7^ VV-ova. Ovaries from infected mice were harvested 4 days later and homogenized in 5-10mL PBS. Serial dilutions were made and added in duplicate onto 24-well plates containing 1.25x10^5^ Vero cells seeded the day before. Viral titer in ovaries was determined by counting plaques and back calculating the number of infectious vaccinia particles per ovary pair.
(TIF)
