# Supplementary figures and images for: Anti-CD8 monoclonal antibody-mediated depletion alters the phenotype and behavior of surviving CD8+ T cells
Source: PLoS One. 2019 Feb 8;14(2):e0211446. doi: 10.1371/journal.pone.0211446 (PMC6368275; doi:10.1371/journal.pone.0211446)

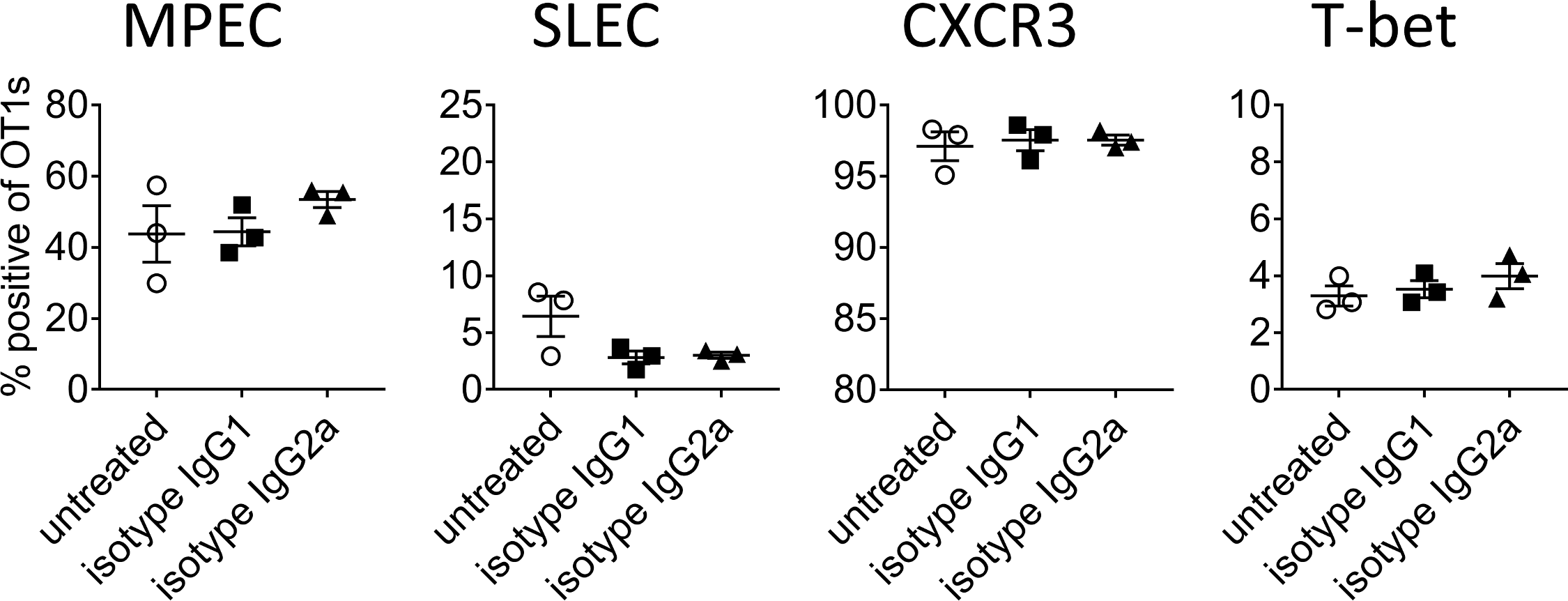

Supplement: S1 Fig — 106 CD45.1+ OT1 T cells were transferred i.v. into CD45.2+ C57BL/6 mice and the next day a high dose (500μg) of isotype control antibody was administered i.p. The mice were immunized the next day and splenocytes harvested 7 days later. (TIF) [file pone.0211446.s001.TIF]

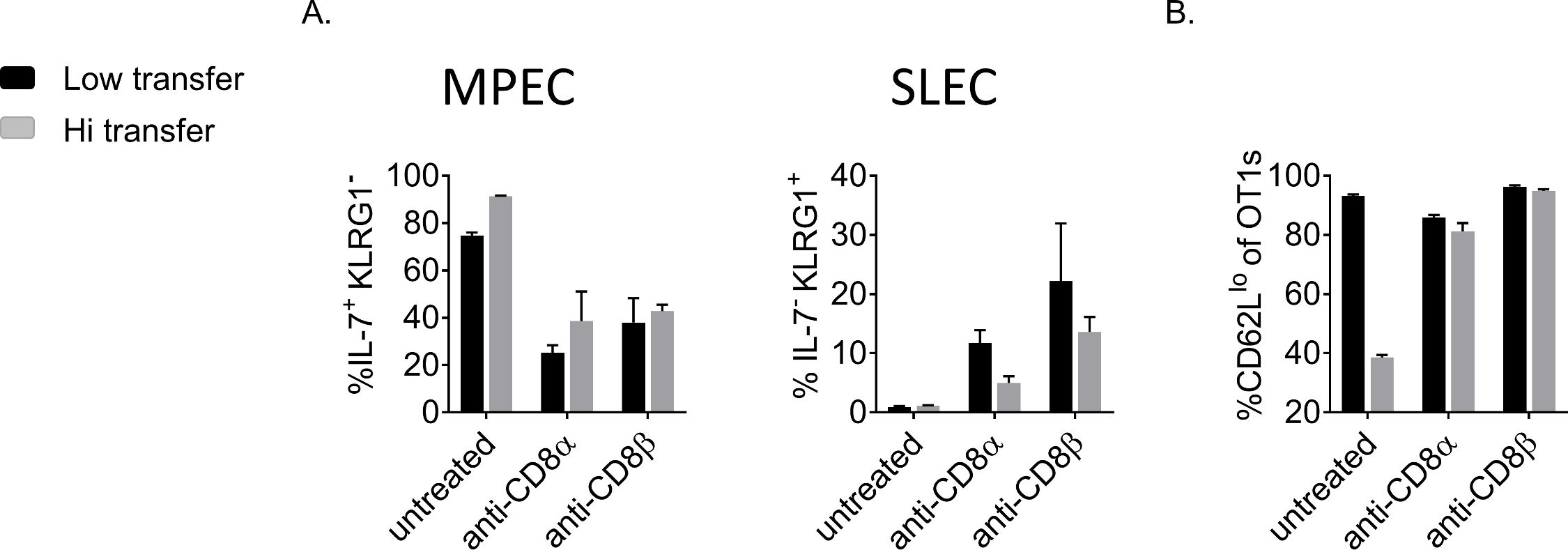

Supplement: S2 Fig — (A-B) Either 5x10^4^ or 106 CD45.1+ OT1 T cells were transferred i.v. into CD45.2+ C57BL/6 mice and the next day a high dose (500μg) of anti-CD8α or –β was administered or no mAb given as a comparison. The mice were immunized the next day and splenocytes harvested 7 days later. (A) The percentage of OT1 T cells that are MPEC (IL-7Rα+ KLRG1-) or SLEC (IL-7Rα- KLRG1+) phenotype. (B) The percentage of OT1 T cells that have downregulated CD62L. Note that anti-CD8 mAb treatment perturbs normal difference seen between the low vs hi precursor frequency. (TIF) [file pone.0211446.s002.TIF]

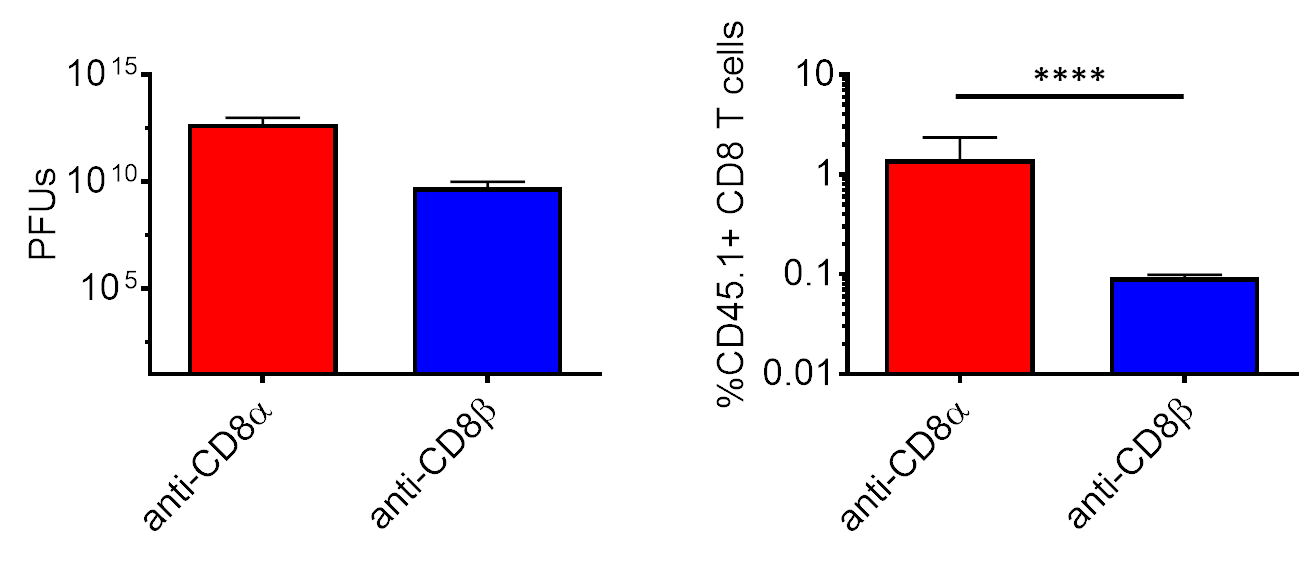

Supplement: S3 Fig — 106 CD45.1+ OT1 T cells were transferred i.v. into CD45.2+ C57BL/6 mice and the next day a high dose (500μg) of either anti-CD8α or -β was administered i.p. The mice were immunized the next day and allowed to rest for 62 days before infection with 107 VV-ova. Ovaries from infected mice were harvested 4 days later and homogenized in 5-10mL PBS. Serial dilutions were made and added in duplicate onto 24-well plates containing 1.25x10^5^ Vero cells seeded the day before. Viral titer in ovaries was determined by counting plaques and back calculating the number of infectious vaccinia particles per ovary pair. (TIF) [file pone.0211446.s003.tif]
